# Real-Time Global Ionospheric Map and Its Application in Single-Frequency Positioning

**DOI:** 10.3390/s19051138

**Published:** 2019-03-06

**Authors:** Liang Zhang, Yibin Yao, Wenjie Peng, Lulu Shan, Yulin He, Jian Kong

**Affiliations:** 1 School of Geodesy and Geomatics, Wuhan University, 129 Luoyu Road, Wuhan 430079, China; qgzhliang@whu.edu.cn (L.Z.); wjpeng@whu.edu.cn (W.P.); llshan@whu.edu.cn (L.S.); ylhe@whu.edu.cn (Y.H.); 2 Key Laboratory of Geospace Environment and Geodesy, Ministry of Education, Wuhan University, 129 Luoyu Road, Wuhan 430079, China; 3 Chinese Antarctic Center of Surveying and Mapping, Wuhan University, 129 Luoyu Road, Wuhan 430079, China; jkong@whu.edu.cn

**Keywords:** real time, ionospheric model, global ionospheric map, DCB, slant ionospheric delay, PPP, RT-SF-PPP

## Abstract

The prevalence of real-time, low-cost, single-frequency, decimeter-level positioning has increased with the development of global navigation satellite systems (GNSSs). Ionospheric delay accounts for most errors in real-time single-frequency GNSS positioning. To eliminate ionospheric interference in real-time single-frequency precise point positioning (RT-SF-PPP), global ionospheric vertical total electron content (VTEC) product is designed in the next stage of the International GNSS Service (IGS) real-time service (RTS). In this study, real-time generation of a global ionospheric map (GIM) based on IGS RTS is proposed and assessed. There are three crucial steps in the process of generating a real-time global ionospheric map (RTGIM): estimating station differential code bias (DCB) using the precise point positioning (PPP) method, deriving slant total electron content (STEC) from PPP with raw observations, and modeling global vertical total electron content (VTEC). Experiments were carried out to validate the algorithm’s effectiveness. First, one month’s data from 16 globally distributed IGS stations were used to validate the performance of DCB estimation with the PPP method. Second, 30 IGS stations were used to verify the accuracy of static PPP with raw observations. Third, the modeling of residuals was assessed in high and quiet ionospheric activity periods. Afterwards, the quality of RTGIM products was assessed from two aspects: (1) comparison with the Center for Orbit Determination in Europe (CODE) global ionospheric map (GIM) products and (2) determination of the performance of RT-SF-PPP with the RTGIM. Experimental results show that DCB estimation using the PPP method can realize an average accuracy of 0.2 ns; static PPP with raw observations can achieve an accuracy of 0.7, 1.2, and 2.1 cm in the north, east, and up components, respectively. The average standard deviations (STDs) of the model residuals are 2.07 and 2.17 TEC units (TECU) for moderate and high ionospheric activity periods. Moreover, the average root-mean-square (RMS) error of RTGIM products is 2.4 TECU for the one-month moderate ionospheric period. Nevertheless, for the high ionospheric period, the RMS is greater than the RMS in the moderate period. A sub-meter-level horizontal accuracy and meter-level vertical accuracy can be achieved when the RTGIM is employed in RT-SF-PPP.

## 1. Introduction

The use of global navigation satellite systems (GNSSs) has widely increased in fields such as geodesy, navigation, precision agriculture, and hazard monitoring [1,2,3,4]. High-accuracy fields using these applications primarily use dual-frequency positioning, which provides millimeter to centimeter accuracy. Nevertheless, because dual-frequency GNSS receivers are more costly than single-frequency devices, single-frequency applications are more feasible for ordinary users.

To satisfy real-time, low-cost, single-frequency GNSS positioning, precise satellite orbit and clock corrections, as well as ionospheric delay corrections, are necessary. The International GNSS Service (IGS) real-time service (RTS), which provides precise orbit and clock corrections via the Internet, was officially launched on 1 April 2013 [5,6]. Official IGS RTS products are generated by combining RTS products from individual IGS analysis centers (ACs). For these RTS products, the individual RTS product CLK90 and the combined product IGS03 have proved to have the best quality [7].

The representation of RTS corrections is named state-space representation (SSR), which allows different physical errors to be successively separated, modeled, and predicted. As the standard of the Radio Technical Commission for Maritime Services (RTCM) plan, three crucial stages are designed for SSR development. The first step is developing corrections of orbit, clock, and code bias to support real-time dual-frequency precise point positioning (PPP). The second step is developing ionospheric vertical total electron content (VTEC) corrections; this step supports real-time single-frequency precise point positioning (RT-SF-PPP). The third step is developing slant total electron content (STEC), tropospheric corrections, and satellite phase bias, which can enable the application of PPP-RTK, a fast integer ambiguity resolution-enabled PPP technique [8]. Currently, most RTS products of individual ACs, as well as official RTS products, provide the first-stage message. Only one of the individual ACs, the Centre National d’Études Spatiales (CNES), is broadcasting the ionospheric VTEC message, which can be employed by RT-SF-PPP to mitigate ionospheric delay.

Ionospheric delay accounts for most errors in RT-SF-PPP [9], and the performance of RT-SF-PPP is highly dependent on the mitigation of ionospheric delays [8]. There are several methods to eliminate ionospheric interference for RT-SF-PPP. The simplest method is the Klobuchar model, which is provided in the global positioning system (GPS) navigation message. However, the drawback is that only 50–60% of the ionospheric delay can be eliminated [10]. Higher-accuracy ionospheric corrections can be obtained from a satellite-based augmentation system (SBAS), such as the Wide Area Augmentation System (WAAS), the European Geostationary Navigation Overlay Service (EGNOS), and the Quasi-Zenith Satellite System (QZSS). In regions covered by an SBAS, such as North America, Europe, and Japan, a positioning accuracy better than 1 m can be achieved. RTS ionospheric correction has become another option to mitigate ionospheric influence since the CNES real-time VTEC products launched. Nie et al. [8] introduced the application and assessment of CNES real-time ionospheric products. Results showed that CNES real-time ionospheric VTEC products can be used to derive sub-meter-level horizontal and meter-level vertical positioning solutions with RT-SF-PPP. However, as of now, the details of ionospheric modeling by the CNES are still not very clear. As mentioned above, broadcasting a real-time global VTEC message is the next step of SSR development. The generation of the real-time global VTEC product, which is also named the real-time global ionospheric map (RTGIM), requires detailed discussion.

In real-time ionospheric modeling, two methods—Geometry-Free (GF) combined with Carrier-to-Code Leveling (CCL) method and PPP with raw observations method—can be used to derive slant total electron content (STEC) [11]. Traditional ionosphere products, such as the global ionospheric map (GIM) provided by the Center for Orbit Determination in Europe (CODE), are mainly generated on the basis of the CCL method; however, PPP with raw observations to derive STEC has been more popular in recent years [11,12,13] and was also adopted by the CNES and applied to VTEC message generation.

The PPP with raw observations method was adopted by Tu et al. [12] to establish global ionospheric models in post mode. An initial ionospheric result with the support of the IGS RTS orbit and clock was presented. Furthermore, Liu et al. [11] proposed a real-time ionospheric modeling method by a real-time PPP method and established an Australian ionospheric model by using adjusted spherical harmonic functions. However, their ionospheric model is still a regional ionospheric model, which cannot be used by global real-time users.

In this study, we focus on the generation of an RTGIM by using observations from current real-time stations in real-time mode. First, IGS RTS products are introduced in the following section. Then, we introduce the methodology used for estimating differential code bias (DCB), deriving STEC PPP with raw observations, and modeling real-time VTEC, as well as the real-time process. Moreover, we report the experiments that were carried out to verify the methods’ effectiveness. Finally, the quality of RTGIM products was assessed by comparing them with the CODE GIM and carrying out RT-SF-PPP experiments.

## 2. IGS RTS Products

There are 8 real-time IGS individual ACs: the Bundesamt für Kartographie und Geodäsie (BKG), the Centre National d’Etudes Spatiales (CNES), Deutsches Zentrum für Luft und Raumfahrt (DLR), the European Space Operations Centre (ESOC), the GeoForschungsZentrum (GFZ), the GMV Aerospace and Defense (GMV), the Natural Resources Canada (NRCan), and Wuhan University (WUHAN). Correction streams from the IGS’ individual ACs are listed in Table 1. For most ACs, two RTS products with different reference points are available. The reference point is an antenna phase center (APC) or center of mass (CoM). RTS products are generated by ACs and then directly broadcasted to real-time users. Also, as listed in Table 2, ESOC and BKG are two IGS RTS Combination Centers (CCs) that provide IGC01/IGS01, IGS02, and IGS03 products. The combination IGC01/IGS01 is a single epoch combination product that is the result of combining CLK10, CLK16, CLK20, CLK22, CLK53, CLK70, CLK80, and CLK93. IGS02 is a Kalman Filter combination product generated by combining CLK10, CLK16, CLK20, CLK22, CLK53, CLK70, and CLK80. Moreover, another Kalman Filter combination product, IGS03, is the result of combining CLK11, CLK91, CLK20, and CLK80.

Zhang et al. [7] studied the accuracies of 9 RTS streams, namely, IGS01, IGS03, CLK01, CLK15, CLK22, CLK52, CLK70, CLK81, and CLK90. Results showed that the accuracy of orbit products ranges from 3.8 to 7.5 cm for different RTS products, and clock accuracy ranges from 1.9 to 5.6 cm. The individual RTS product CLK90 and combined product IGS03 have been proved to have the best quality. For these overall RTS products, only the products CLK90, CLK91, CLK92, and CLK93, all of which are provided by the CNES, contain a VTEC message. Nie et al. [8] assessed the VTEC products and concluded that sub-meter-level horizontal and meter-level vertical positioning solutions with RT-SF-PPP could be obtained. Thus, in this study, precise orbit and clock products from IGS03 were adopted for the generation of the RTGIM, and the accuracy of the CNES VTEC was used as a reference.

## 3. Methodology

An infinitesimal thin shell is adopted for most of the current VTEC ionospheric models. A VTEC model can be defined as spherical harmonic expansions; the model is widely used, similar to the usage frequency of CODE GIM products and CNES real-time VTEC products. To build a global VTEC model, STEC derived from globally distributed stations is required, and it can be obtained from PPP with raw observations method [13]. Moreover, DCBs of stations are necessary for PPP with raw observations method and can be estimated in the post-PPP process [12]. In this section, the derivation of STEC from PPP with raw observations method and the estimation of DCB with the PPP method are presented. Afterward, global ionospheric spherical harmonic modeling is described. Finally, data processing for generating an RTGIM is briefly introduced.

### 3.1. PPP with Raw Observations

The linearized equations for PPP with raw observations can be expressed as [11,13,14]

(1) $$\begin{array}{cc} \Delta P_{r,f}^{s} & =\vec{e_{r}^{s}}\cdot \Delta \vec{x}+c\hat{\Delta t_{r}}+\mu_{f}{\hat{I}}_{r,1}^{s}+m_{r}^{s}T_{r}+\epsilon_{P}\\ \Delta L_{r,f}^{s} & =\vec{e_{r}^{s}}\cdot \Delta \vec{x}+c\hat{\Delta t_{r}}-\mu_{f}{\hat{I}}_{r,1}+m_{r}^{s}T_{r}+\lambda_{f}^{s}{\hat{N}}_{r,f}^{s}+\epsilon_{L} \end{array}$$

where the superscript *s* and subscripts *r* and *f* denote specific satellites, receivers, and frequencies, respectively; $\Delta P_{r,f}^{s}$ and $\Delta L_{r,f}^{s}$ are the observed-minus-computed (O-C) raw code and phase observations with corrections (tidal effects, antenna PCO/PCV, phase wind-up, etc.); $\vec{e_{r}^{s}}$ is the unit vector from satellite to receiver; $\Delta \vec{x}=\left[\Delta x,\Delta y,\Delta z\right]$ is the station increment vector; *c* denotes the speed of light; $\Delta {\hat{t}}_{r}$ is the clock offset for the receivers; $\mu_{f}={\left(\lambda_{f}^{s}/\lambda_{1}^{s}\right)}^{2}$ is the frequency-related factor; $I_{r}^{s}$ is the ionospheric delay on L1 frequency; $T_{r}$ is the site-specific zenith tropospheric delay; $m_{r}^{s}$ is the mapping function; $\lambda_{f}^{s}$ is the wavelength of the frequency *f*; ${\hat{N}}_{r,f}^{s}$ is the float phase ambiguity absorbing receiver and satellite phase hardware delays; $\epsilon_{p}$ and $\epsilon_{L}$ are the observation noises, multipath effects, and other unmodeled errors for code and phase observations, respectively. Satellite orbit and clock errors are corrected with IGS03 RTS products. The symbols with a ^ on top denote reparametrized estimable parameters [11]:

(2) $$\begin{array}{cc} \Delta {\hat{t}}_{r} & =\Delta t_{r}+\left(\frac{\mu_{2}}{\mu_{2}-1}b_{r,1}-\frac{1}{\mu_{2}-1}b_{r,2}\right),\\ {\hat{I}}_{r,1}^{s} & =I_{r,1}^{s}-\frac{c}{\mu_{2}-1}\left(DCB_{r}+DCB^{s}\right),\\ {\hat{N}}_{r,f}^{s} & =N_{r,f}^{s}+\frac{c}{\lambda_{f}^{s}\left(\mu_{2}-1\right)}\left[\left(\mu_{f}+1\right)\left(b_{r,2}+b_{2}^{s}\right)-\left(\mu_{f}+\mu_{2}\right)\left(b_{r,1}+b_{1}^{s}\right)\right] \end{array}$$

where $\Delta {\hat{t}}_{r}$ is the receiver clock containing code hardware delays; ${\hat{I}}_{r,1}^{s}$ is the ionospheric delay biased by the satellite and receiver DCBs, where $DCB^{s}=b_{1}^{s}-b_{2}^{s}$ and $DCB_{r}=b_{r,1}-b_{r,2}$; ${\hat{N}}_{r,f}^{s}$ is the float phase ambiguity containing code and phase hardware delays. More details of the method for deriving STEC by PPP with raw observations can be found in Liu et al. [11], Tu et al. [12], Zhang et al. [13].

As per the raw observation equations (Equation (1)), if DCBs are known, the unknown parameters are the station coordinate increment vectors, the slant ionospheric delay, and the float phase ambiguity. Satellite DCB can be obtained from the CODE. However, DCBs of some stations are unknown. Thus, DCBs of stations are estimated before applying real-time PPP.

### 3.2. DCB Estimation by PPP Method

Differential Code Biases (DCBs) are the systematic errors or biases between two GNSS code observations at the same or different frequencies. To estimate receiver DCBs, Equation (1) can be rewritten as follows:

(3) $$\begin{array}{cc} \Delta P_{r,f}^{s} & =\vec{e_{r}^{s}}\cdot \Delta \vec{x}+c\hat{\Delta t_{r}}+\mu_{f}I_{r,1}^{s}-\frac{c\mu_{f}}{\mu_{2}-1}\left(DCB_{r}+DCB^{s}\right)+m_{r}^{s}T_{r}+\epsilon_{P}\\ \Delta L_{r,f}^{s} & =\vec{e_{r}^{s}}\cdot \Delta \vec{x}+c\hat{\Delta t_{r}}-\mu_{f}I_{r,1}^{s}+\frac{c\mu_{f}}{\mu_{2}-1}\left(DCB_{r}+DCB^{s}\right)+m_{r}^{s}T_{r}+\lambda_{f}^{s}{\hat{N}}_{r,f}^{s}+\epsilon_{L} \end{array}$$

In Equation (3), the satellite DCB is known; if the ionosphere prior constraint is added, the receiver DCB can be estimated in the PPP process [12]. The CODE GIM was used as the prior constraint.

For the stations without P1 and P2 observations, C1 and C2 were used instead. Thus, satellite P1-C1 and P2-C2 DCBs were added during the PPP process [15].

### 3.3. Spherical Harmonic Function Model

In Equations (1) and (2), the ionospheric delay $I_{r,1}^{S}$ can be estimated in the PPP process. Based on the infinitesimal thin shell hypothesis, VTEC can be obtained from the ionospheric delay $I_{r,1}^{S}$ [16]:

(4) $$\begin{array}{cc} VTEC & =MF(z)STEC\\ MF(z) & =cos\left(arcsin\left(\frac{R}{R+H}sin\left(\alpha z\right)\right)\right)\\ STEC & =\frac{F_{f}^{2}}{40.28}I_{r,f}^{s} \end{array}$$

where MF(z) is the ionospheric mapping function; *z* is the satellite elevation angle; *R* is the Earth’s radius; *H* is the attitude of the ionosphere thin shell. *H* and α can be set by the users. Here, *H* and α were set to 506.7 km and 0.9782.

The spherical harmonic function, which is often used to express global ionospheric models, is expressed as follows [3]:

(5) $$VETC\left(\phi ,\lambda \right)=\sum_{n=0}^{N}\sum_{m=0}^{n}P_{n}^{m}\left(sin\phi \right)\left(A_{n}^{m}cos\left(m\lambda \right)+B_{n}^{m}sin\left(n\lambda \right)\right),m\leq n\leq N$$

where ϕ is the geocentric latitude of the ionospheric pierce point (IPP), and λ is the solar-fixed longitude of the IPP. *N* is the degree of spherical harmonic function and was set to 12 in this study; $P_{n}^{m}$ is the regularization Legendre series; $A_{n}^{m}$ and $B_{n}^{m}$ are the spherical harmonic coefficients for estimation. VTEC is vertical total electron content (TEC) at the IPP.

### 3.4. Real-Time Data Process

**Figure 1:**
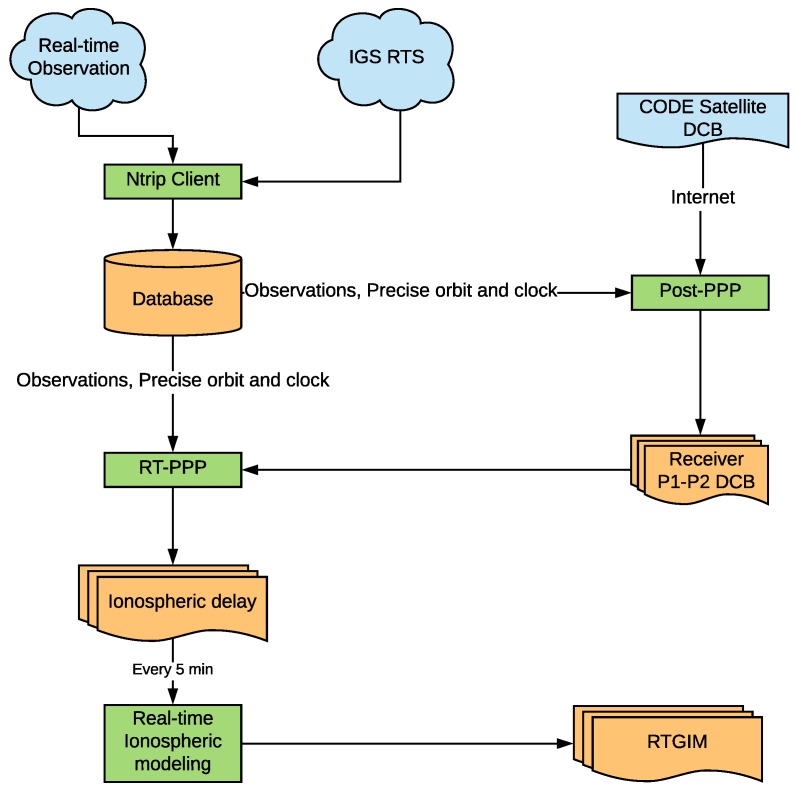
Flowchart of RTGIM generation.

The RTGIM generation process is shown in Figure 1. To generate GIM in real time, receiving and decoding the real-time streams of RTS and observations is the first step. An RTCM networked transport via an Internet protocol (NTRIP [17]) client, SGGNtrip, is used. SGGNtrip was developed at the School of Geodesy and Geomatics (SGG), Wuhan University. The client can receive, decode, and store (in a database) RTCM messages, such as observations, broadcast ephemeris, orbit and clock corrections, and other RTCM messages. The client is designed based on a database, which means that once real-time data are stored in the database, the data can be accessed efficiently for different applications, including the PPP process, real-time ionospheric monitoring, or DCB estimation.

Orbit and clock, as well as global observations streams, are necessary for real-time PPP. However, orbit and clock streams are always later than observations because of product latency [7]. In real-time PPP, once an observation of an epoch is received, the epoch is processed. The newest received corrections of orbit and clock are applied to observation time.

DCBs of stations are estimated every day by a post-PPP process. Monthly P1-C1 and P1-P2 DCBs of satellites from the CODE are downloaded every month and used in the post-PPP process. Because DCB is stable for several days, the estimated DCB value is not employed until the difference from the used DCB is too large.

STEC derived from the real-time PPP process is also saved in a database. The modeling process queries the most recent 1-h STEC data from the database every 5 min. Afterward, VTEC is converted by a mapping function from STEC, and the spherical harmonic coefficients are estimated. RTGIM products are generated with a resolution of 5 (longitude) × 2.5 degrees (latitude), which is the same as the resolution of the CODE GIM products.

In the global ionospheric modeling process, outlier detection is a crucial factor that affects ionospheric accuracy. There are two outlier detection means in the generation of an RTGIM. The first is applied in the real-time PPP process. Coordinates are estimated in real time, and the reference coordinates of stations are known beforehand. Once the difference is greater than 10 cm on the horizontal component and 25 cm on the vertical component, the epoch is regarded as an invalid solution, and the STEC at this epoch is marked as an outlier. The second is in global ionospheric modeling. If a model residual of STEC is greater than 3 times the STD, it is also marked as an outlier, and twice iteration will be adopted for spherical harmonic coefficient estimation.

Actual computation time and product latency are crucial for RTGIM products. There are three time-consuming procedures in RTGIM generation: real-time observation receiving, PPP computation, and spherical harmonic coefficient estimation. In general, the latency of a real-time observation is about 2–3 s; PPP computation costs less than 3 s for about 80 stations. The most time-consuming procedure is the estimation of spherical harmonic coefficients, which costs about 63 s for a 12-degree spherical harmonic function. Thus, the overall time-cost is about 70 s for each product update.

## 4. Experiments and Results

In this section, experiments are divided into two parts. First, experiments of DCB estimation, static PPP with raw observations, and global ionospheric modeling are presented to verify the effectiveness of the RTGIM generation method. Second, the quality of RTGIM products is assessed.

### 4.1. Experiments for Algorithm Validation

Static PPP with raw observations is a crucial step that is needed to obtain STEC. DCBs of satellites and stations are necessary for the PPP process. DCB estimation using the PPP method was first validated. Afterward, to validate the performance of static PPP with raw observations, global distribution station experiments were carried out. Finally, the modeling method was evaluated in different ionospheric activity periods.

#### 4.1.1. Test of DCB Estimation

DCBs are necessary for estimating slant ionospheric delay in real-time PPP. Jin et al. [16] proposed a DCB estimation method that was based on the CCL method and global ionospheric modeling. Experimental results showed that DCBs of station accuracy were better than 0.3 ns for most stations.

To investigate the accuracy of the DCB estimation using the PPP method, experiments using globally distributed stations were carried out. Observation data from 16 global distribution IGS stations were collected from day of year (DOY) 121 (1 May) to DOY 151 (31 May), 2018. The orbit and clock obtained from IGS03 were fixed, and satellites P1-P2, P1-C1, and P2-C2 from May 2018 (provided by the CODE) were adopted in the PPP process. The prior information constraint of the ionospheric delay was also required for DCB estimation. Thus, the CODE GIM from the same day was used. Finally, DCBs, tropospheric parameters, coordinates, and ambiguities were estimated.

**Figure 2:**
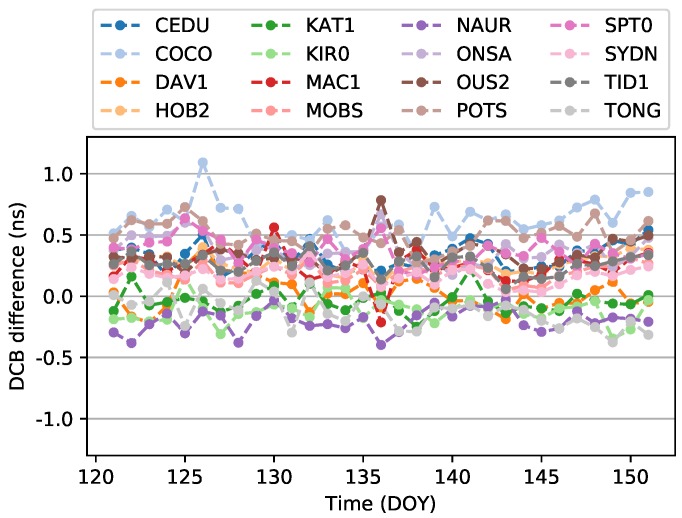
DCB difference from the CODE values in May 2018.

The P1-P2 DCB differences from the CODE values of 16 stations from DOY 121 to DOY 151, 2018, are shown in Figure 2. As shown in the figure, the vertical axis stands for the difference, and the horizontal axis stands for the day of year in 2018. For all stations in May 2018, the DCB difference varies between 0 and 0.8 ns, except for the station COCO, which reaches 1.2 ns on DOY 126. The best three stations are KAT1, DAV1, and TONG, which are less than 0.3 ns in that month. For the one-month period, the variation in DCBs for most stations is less than 0.5 ns.

**Figure 3:**
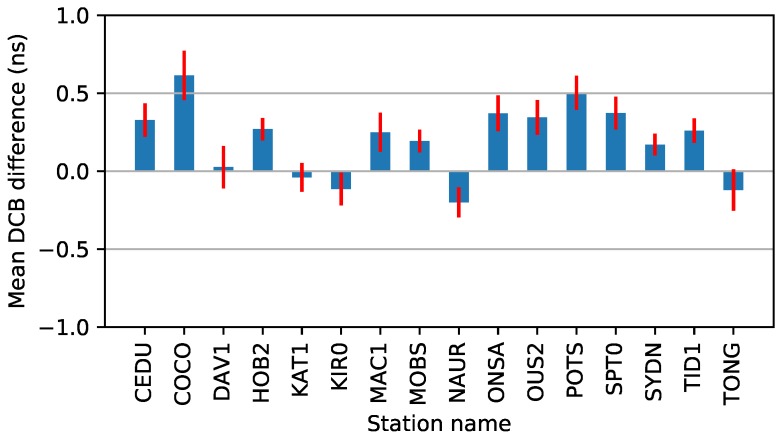
Mean of the DCB difference from the CODE values in May 2018. The mean bias and STD of the DCB differences are in blue and red colors. The average of the mean and STD values is 0.2 and 0.1 ns, respectively.

Moreover, the mean bias and standard deviation (STD) of the DCB differences are presented in Figure 3. As we can see, the largest value is 0.6 ns for the station COCO, and the smallest value is 0.02 ns for the station DAV1. The average DCB difference of all stations is 0.2 ns. For all stations, most of the STD values are similar, with values less 0.2 ns. The average STD is 0.1 ns. Compared with the results of Jin et al. [16], DCB estimation with the PPP method has similar accuracy.

#### 4.1.2. Test of Static PPP

To investigate the accuracy of real-time static PPP as determined with raw observations, 30 stations from DOY 135 (May 15), 2018, were collected for the experiment. The orbit and clock from IGS03 were employed. P1-P2 and P1-C1 DCBs of satellites, as well as the estimated P1-P2 DCBs of stations, were fixed in the PPP process. More detailed static PPP settings are provided in Table 3. The IGS station coordinates from the IGS weekly solution or from SOPAC were used as a reference.

**Figure 4:**
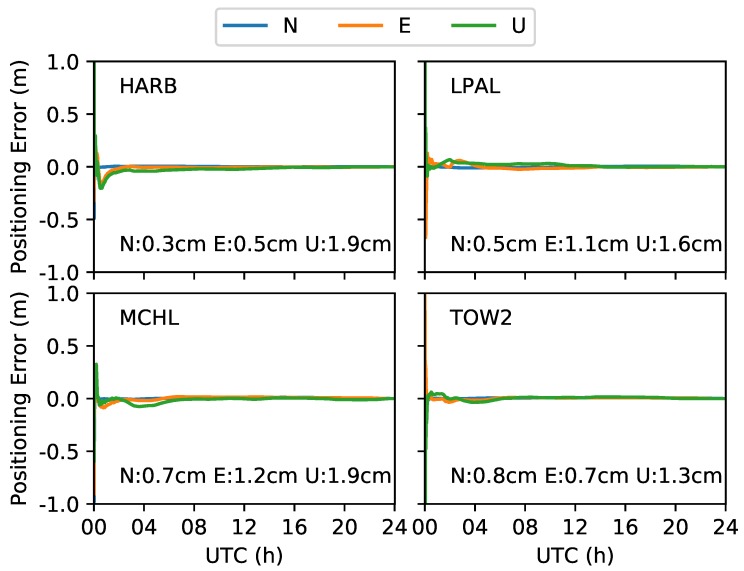
Positioning errors of 4 stations.

The positioning errors of 4 stations are shown in Figure 4. As shown in the figure, the accuracy of the station HARB for the three components converges easily after a short time. For the north component, accuracy reaches 10 cm in 10 min; for the east and up components, accuracy reaches 10 cm in about 1 h. Finally, the three components can reach about 1 cm.

The convergence time differs for each station in the figure. For stations HARB and TOW2, the positioning errors for the east and up components are relatively better than those from the stations LPAL and MCHL. For the north components, the four stations can achieve an accuracy of 10 cm in 20 min.

**Figure 5:**
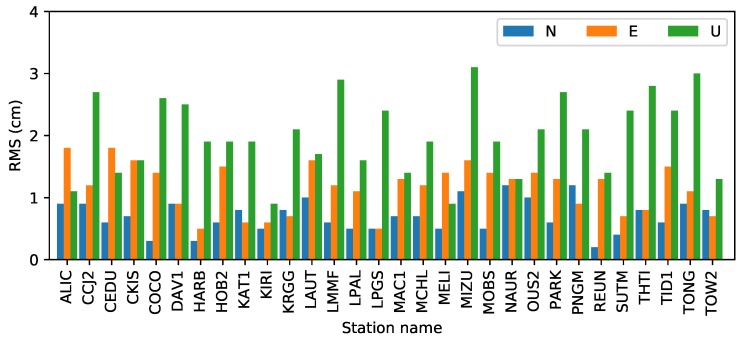
Positioning errors of real-time static PPP experiments. The mean RMS values are 0.7, 1.2, and 2.1 cm in the north, east, and west components, respectively.

The root-mean-square (RMS) errors in the three components from 30 stations are shown in Figure 5. As we can see, the maximum RMS values are 1.2, 1.8, and 3.1 cm in the north, east, and up components. The minimum values are less than 5 mm on the horizontal and 1 cm on the vertical. The mean values in the three components are 0.7, 1.2, and 2.1 cm. Finally, the north and up values represent the best and worst components, respectively.

#### 4.1.3. Test of Ionospheric Modeling

**Figure 6:**
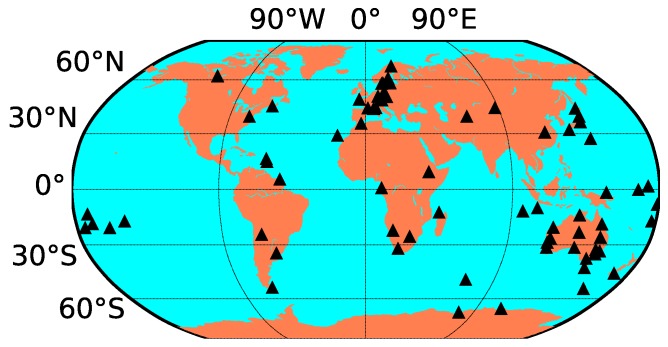
Globally distributed stations for global ionospheric modeling.

To conduct a global ionospheric model, STEC values derived from global stations by the PPP method are required. Data from real-time stations on DOY 135, 2018, were used for global ionospheric modeling. As shown in Figure 6, the real-time station distribution is uneven. The highest-density stations are in Western Europe and Oceania, with more than half of all stations distributed from within these two regions. In the Eastern Asian region, there are more stations than in North America and Northern Africa. Moreover, few stations are distributed in the Middle East, South Asia, and the Arabian Peninsula. The uneven spread of the stations will cause an uneven IPP distribution, which can lead to negative values in blank areas. To avoid negative values, a prior constraint was added to the modeling process. The RTGIM of the previous day for the same time of day was used as the constraint, and the VTEC accuracy at each grid point was set to 10 TEC units (TECU).

**Figure 7:**
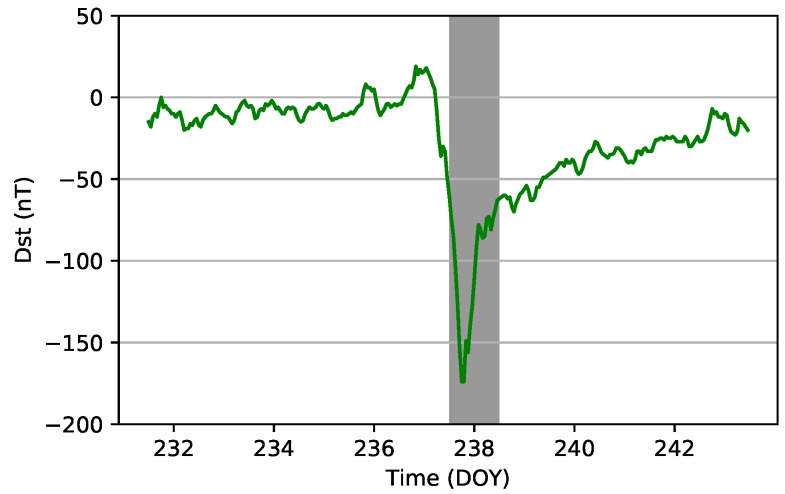
Dst index around DOY 238, 2018.

To investigate the accuracy of ionospheric modeling in high and moderate ionospheric periods, STEC of DOY 135 and DOY 238, 2018, was used. As shown in Figure 7, the disturbance storm time (Dst) index on DOY 238, 2018, reached −174 nT, which represents the high ionospheric active period. Meanwhile, the Dst index on DOY 135, 2018, is greater than −13, which represents the moderate ionospheric period.

**Figure 8:**
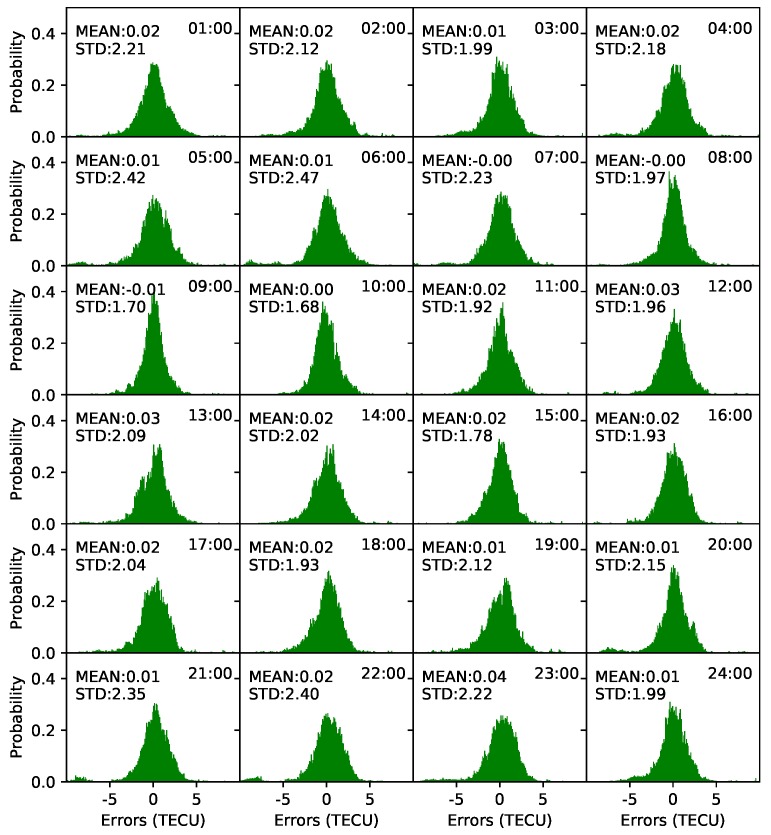
Frequency distribution histograms of model residuals on DOY 135, 2018.

**Figure 9:**
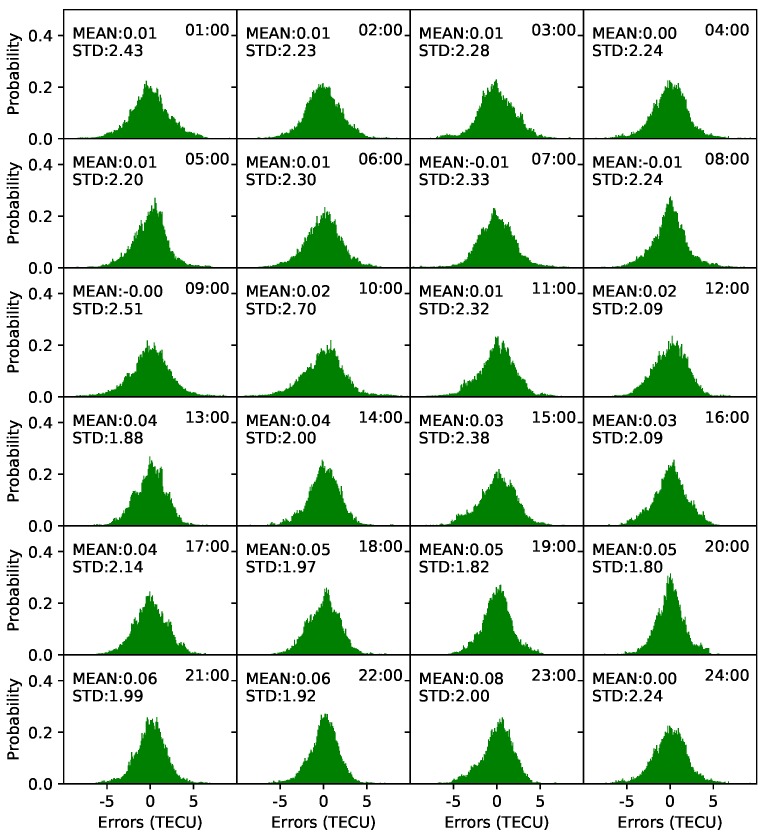
Frequency distribution histograms of model residuals on DOY 238, 2018.

Spherical harmonic coefficients were estimated every 5 min. One-hour STEC values before the modeling epoch were used. Frequency distribution histograms of model residuals were plotted every 1 h to verify the accuracy of the models. As shown in Figure 8, the distributions of the residuals are within normal expectations. Almost all the mean values are less than 0.02 TECU, and the STD values are less than 2.5 TECU. The minimum STD value is 1.68 TECU at 10:00 UT, and the maximum STD value is 2.47 TECU at 6:00 UT. The mean of the STD values is 2.07 TECU. In contrast, the residual distribution on DOY 238, 2018, is presented in Figure 9. As shown in the figure, the residual distributions are also within normal expectations. The mean of the residuals varies from 0 to 0.08 TECU. In particular, from 13:00 UT to 23:00 UT, the mean of the residuals is greater than that from 1:00 UT to 12:00 UT. The minimum STD value is 1.8 TECU at 20:00 UT, and the maximum STD is 2.7 TECU at 10:00 UT. The mean of the STD values is 2.17 TECU, which is slightly greater than the value on DOY 135.

**Figure 10:**
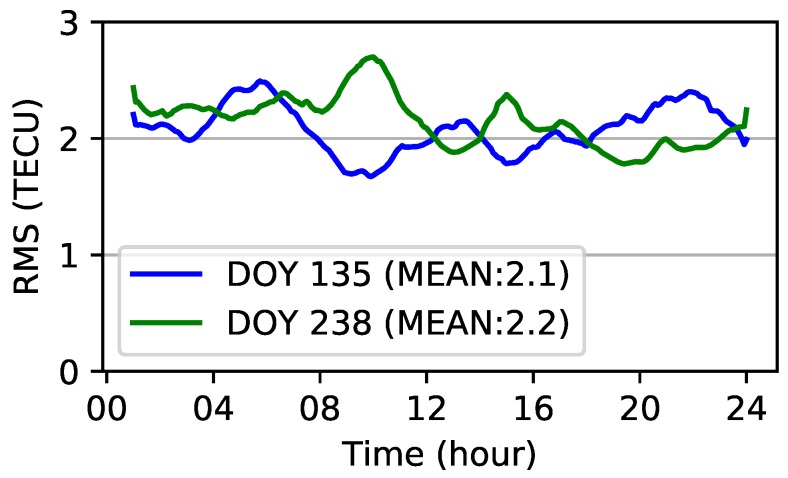
RMSE values of modeling residuals on DOY 135 and DOY 238, 2018.

Moreover, RMS values in 5 min intervals in the two days studied are presented in Figure 10. As shown in the figure, the RMS values of the modeling residuals vary from 1.6 to 2.5 TECU on DOY 135. Meanwhile, the RMS values are in the range of 1.8–2.7 TECU. The mean of the RMS values is 2.1 and 2.2 TECU for DOY 135 and DOY 238, respectively. The modeling accuracy on DOY 135 is slightly better than that on DOY 238.

### 4.2. Quality Assessment of RTGIM Products

The quality assessment of RTGIM contains two aspects: first, the accuracy compared with the reference product; second, the positioning accuracy when the RTGIM product is adopted.

#### 4.2.1. Comparison with CODE GIM Products

**Figure 11:**
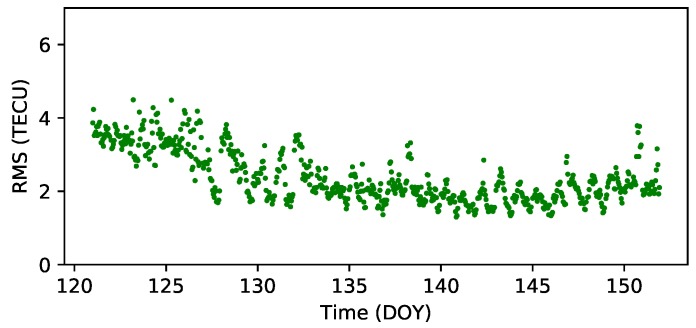
Time series of RMS for RTGIM from DOY 121 to DOY 151, 2018. The mean and STD values are 2.4 and 0.7 TECU, separately.

To assess the accuracy of RTGIM products, the CODE GIM was set as the reference, and the differences between each grid points relative to the CODE GIM were calculated. The global RMS values for all grid points at each epoch from DOY 121 to DOY 151 are presented in Figure 11. As shown in the figure, the minimum value is 1.3 TECU at 20:00 UT on DOY 140, and the maximum value is 4.9 TECU at 5:00 UT on DOY 123. The mean value is 2.4 TECU, and the STD value is 0.7 TECU.

**Figure 12:**
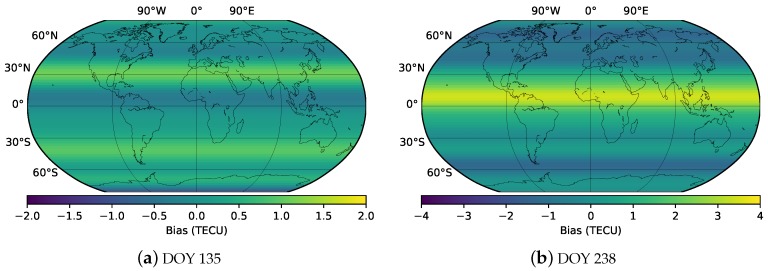
Bias of different latitudes at 12:00 UT on DOY 135 (**a**) and DOY 238 (**b**), 2018.

**Figure 13:**
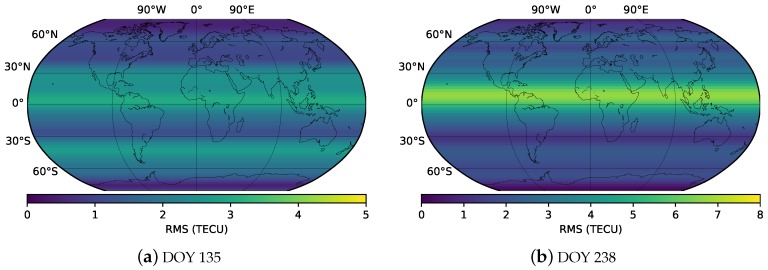
RMS values of different latitudes at 12:00 UT on DOY 135 (**a**) and DOY 238 (**b**), 2018.

To further verify the accuracy of the RTGIM in different ionospheric activity periods, bias and RMS for different latitudes at 12:00 UT on DOY 135 and DOY 238 are shown in Figure 12 and Figure 13, respectively. As shown in the figures, the bias and RMS of the RTGIM share similar spatial features. For the bias map at 12:00 UT on DOY 135, the bias values vary from −0.87 to 1.11 TECU, and the RMS values vary from 0.45 to 3.06 TECU. The maximum bias is around 30 degrees north latitude, and the maximum RMS is around 5 degrees north latitude. At 12:00 UT on DOY 238, the bias values vary from −1.33 to 3.45 TECU, and the RMS values vary from 0.11 to 6.73 TECU. The maximum bias is around 10 degrees north latitude, and the maximum RMS is around 5 degrees north latitude. The RMS values of the global grid points vary from 1.8 to 3.1 TECU for 12:00 UT on DOY 135 and DOY 238, 2018, respectively. By comparing RMS values between the two days, we can see that the accuracy during the moderate ionospheric activity period is better than that during the high activity period.

In the study by Nie et al. [8], the CNES VTEC products were assessed by comparing them with the IGS final GIM products. The ranges of the bias and RMS for the different latitudes are listed in Table 4. As shown in the table, two different degrees of spherical harmonic functions were used for period 1 and period 2. For the 12-degree function in period 2, the bias at different latitudes varies from −2.82 to 0.06 TECU, and the RMS for the CNES product varies from 0.97 to 3.01 TECU, which is similar to the RTGIM in the moderate period. For the high ionospheric activity period, the accuracy is slightly worse than that in the quiet period of the RTGIM.

#### 4.2.2. Single-Frequency PPP Performance in User Domain

A real-time ionospheric model is also necessary for RT-SF-PPP. To investigate positioning accuracy when adopting the RTGIM in the user domain, RT-SF-PPP experiments with 31 globally distributed stations were conducted. Please note that RT-SF-PPP was simulated by processing the GPS data in post mode. Orbit and clock corrections from IGS03 were applied to correct orbit and clock errors, and ionospheric delay errors were corrected by RTGIM. Tropospheric errors were corrected by the Saastamoinen model [18]. The RT-SF-PPP settings are listed in Table 5.

**Figure 14:**
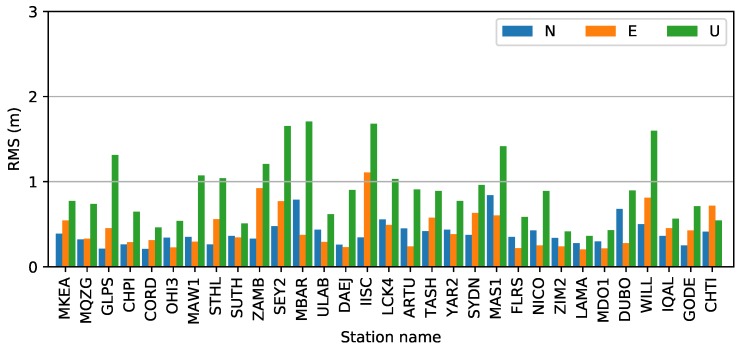
RMS values of RT-SF-PPP for 31 IGS stations on DOY 135. The average RMS values in the north, east, and up components are 0.46, 0.52, and 1.01 m, respectively.

The precise coordinates of the stations from the IGS weekly SINEX solution or from SOPAC were used as the reference. Positioning errors of RT-SF-PPP are shown in Figure 14. As shown in the figure, positioning accuracy of the horizontal and vertical components can reach the sub-meter level and meter level. The average RMS values in the north, east, and up components are 0.46, 0.52, and 1.01 m, respectively. For the up component, the RMS values vary from 0.41 to 1.7 m. Six stations, namely, GLPS, SEY2, MBAR, IISC, MAS1, and WILL, are greater than 1.2 m for the up component. RT-SF-PPP experiments with CNES VTEC products were also carried out by Nie et al. [8]. The average RMS values of 31 globally distributed stations are 0.492, 0.479, and 0.97 m in period 2 when the 12-degree spherical harmonic function is adopted. The accuracy of RT-SF-PPP with the RTGIM is close to that of the CNES VTEC products.

**Figure 15:**
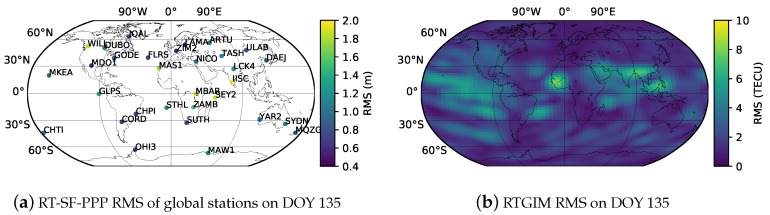
Global maps of RT-SF-PPP and RTGIM accuracies.

To verify geographical corrections of positioning accuracy and RTGIM accuracy, RT-SF-PPP 3-D RMS values of global stations are plotted on the global map, as well as RTGIM ionospheric RMS values on DOY 135, which are presented in Figure 15. According to the global map of RMS on DOY 135, the accuracy is uneven for different regions. The RMS varies from 0.3 to 7.9 TECU. For most regions of the world, the RMS is less than 3 TECU. Nevertheless, for some regions, such as the Western Pacific, Northern Africa, Southern Asia, the Arabian Peninsula, and the Northern Indian Ocean, the RMS values are greater than those in other regions. As presented in Figure 15a, the 3-D RMS values of the global stations vary from 0.5 to 2 m. There are 6 stations with an accuracy worse than 1.4 m, namely, IISC, MBAR, GLPS, SEY2, WILL, and MAS1. As we can see from the two maps, the 6 stations are in low-accuracy regions. Thus, the positioning accuracy is obviously affected by the accuracy of the ionospheric product.

## 5. Conclusions

Ionospheric delay correction is necessary for RT-SF-PPP. In this study, the generation of an RTGIM based on IGS RTS products and real-time IGS stations is proposed. Experiments were carried out to validate the algorithm and assess product quality.

Generating a real-time global ionospheric map requires three crucial steps: DCB estimation with the PPP method, STEC estimation using PPP with raw observations method, and global ionospheric modeling. To assess the DCB estimated by the PPP method, one month’s data from 16 stations were collected and used to estimate DCBs for each day between DOY 121, 2018, and DOY 151, 2018. Experimental results show that the accuracy of DCBs for the stations is about 0.2 ns, and the average STD value is 0.1 ns; these values represent an accuracy that is similar to those derived using the CCL DCB estimation method [16]. STEC was derived from static PPP with raw observations. To assess the performance of the PPP process, 30 globally distributed stations, as well as IGS03 RTS products, were used for positioning experiments. Results show that the mean accuracies for the stations are 0.7, 1.2, and 2.1 cm in the three components (north, east, and up, respectively). To assess the VTEC modeling accuracy for different ionospheric activity periods, VTEC values derived from real-time stations on DOYs 135 and 238 were used in the modeling tests. Results show that the STDs of the model residuals are 2.07 and 2.17 TECU on average in the moderate and high ionospheric periods, respectively. The modeling accuracy in the high activity period is slightly greater than that in the moderate period.

Product quality was assessed from two aspects. First, the differences from the reference for one month’s data (from DOY 121 to DOY 151, 2018) were evaluated. Second, RT-SF-PPP experiments were performed by using 31 globally distributed IGS stations. With the CODE GIM products as references, the RMS values of different grid points at each epoch vary from 1.3 to 4.9 TECU. The mean value is 2.4 TECU, and the STD is 0.7 TECU. To further verify the RTGIM in the high ionospheric activity period, the products on DOY 238 were evaluated. Bias and RMS were calculated for different latitudes. The RMS varies from 0.45 to 3.06 TECU on DOY 135 and from 0.45 to 3.06 TECU on DOY 238. The accuracy in the high ionospheric period is lower than that in the moderate period. The RTGIM shares an approximate accuracy with the CNES VTEC product. The RT-SF-PPP experiments show that the mean RMS values in the north, east, and up components are 0.46, 0.52, and 1.01 m. The performance is also close to that of the CNES VTEC product. The poor-performing stations are in the poor RTGIM accuracy regions, where there is low coverage by the reference stations.

Therefore, it is concluded that it is feasible to generate real-time global ionospheric map products based on IGS RTS products and real-time IGS stations. Accuracy at the sub-meter level and meter level on horizontal and vertical components, respectively, can be achieved for RT-SF-PPP.

## Figures and Tables

**Table 1:** Correction streams from the IGS RTS by individual ACs.

AC	Description	Mountpoint Com/APC
BKG	GPS + GLONASS RT orbits and clocks using IGU orbits	CLI00/CLK10
	GPS + GLONASS RT orbits and clocks using IGU orbits	CLK01/CLK10
CNES	GPS + GLONASS orbits and clocks	CLK90/CLK91
	GPS + GLONASS + Galileo + Beidou orbits and clocks	CLK92/CLK93
DLR	GPS + GLONASS + Galileo + Beidou RT orbits and clocks	CLK20/CLK21
ESOC	RT orbits and clocks using NRT batch orbits every 2 h	
	which are based on IGS Batch hourly files	CLK50/CLK51
	RT orbits and clocks using NRT batch orbits every 2 h	
	which are based on RINEX files generated from the RT stream	CLK52/CLK53
GFZ	RT orbits and clocks and IGU orbits	CLK70/CLK71
GMV	GPS + GLONASS orbits and clocks based on NRT orbit solution	CLK81/CLK80
NRCan	GPS orbits and clocks using NRT batch orbits every hour	-/CLK22
WUHAN	GPS orbits and clocks based on IGU orbits	CLK15/CLK16

**Table 2:** Combined correction streams by IGS RTS Combination Centers.

Center	Description	Mountpoint
		Com/APC
ESOC	GPS-only combination—epoch-wise approach	IGC01/IGS01
BKG	GPS-only combination—Kalman filter approach	-/IGS02
	GPS + GLONASS combination—Kalman filter approach	-/IGS03

**Table 3:** Static PPP experimental settings.

Options	Settings
Constellation	GPS
Measurement	raw observations
Positioning mode	static
Frequencies	L1, L2
Sampling rate	30 s
Elevation mask	$7^{\circ }$
Tropospheric zenith hydrostatic	Saastamoinen
Tropospheric zenith wet delay	initial model + estimated (random walk process)
Tropospheric mapping function	GMF
Phase wind-up	Corrected
Sagnac effect, relativistic effect	Corrected with IGS absolute
Receive clock	Estimated
Station coordinates	Estimated

**Table 4:** Accuracy of the CNES VTEC and RTGIM compared with the reference (unit: TECU).

Product	Bias	RMS
CNES Period 1 (6 degrees)	−3.15 to −0.03	1.06–4.91
CNES period 2 (12 degrees)	−2.82 to 0.06	0.97–3.01
RTGIM quiet ionospheric period	−0.87 to 1.11	0.45–3.06
RTGIM high ionospheric period	−1.33 to 3.45	0.11–6.73

**Table 5:** Single-frequency precision point positioning settings.

Option	Setting
Constellation	GPS
Positioning mode	Kinematic
Orbit	Broadcast ephemeris + IGS03
Clock	Broadcast ephemeris + IGS03
Measurement	P1 and L1 or C1 and L1
Ionospheric correction	RTGIM
Tropospheric correction	Saastamoinen model
Elevation mask	$7^{\circ }$

## Abbreviations

The following abbreviations are used in this manuscript:

AC	Analysis Center
APC	Antenna Phase Center
BKG	the Bundesamt für Kartographie und Geodäsie
CC	Combination Center
CCL	Carrier-to-Code Leveling
CNES	the Centre National d’Etudes Spatiales
CODE	the Center for Orbit Determination in Europe
CoM	Center of Mass
DCB	Difference Code Bias
DLR	Deutsches Zentrum für Luft- und Raumfahrt
EGNOS	the European Geostationary Navigation Overlay Service
GF	Geometry-Free
GFZ	GeoForschungsZentrum
GPS	Global Positioning System
GMV	GMV Aerospace and Defense
GNSS	Global Navigation Satellite System
GIM	Global Ionospheric Map
IF	Ionosphere-Free
IGS	International GNSS Service
IPP	Ionosphere Pierce Point
NTRIP	Networked Transport of RTCM via Internet Protocol
NRCan	Natural Resources Canada
PPP	Precise Point Positioning
QZSS	the Quasi-Zenith Satellite System
RTS	Real-time Service
RTGIM	Real-time Global Ionospheric Map
RT-SF-PPP	Real-time Single-frequency Precise Point Positioning
RMSE	Root-Mean-Square Error
RTCM	Radio Technical Commission for Maritime Services
SGG	School of Geodesy and Geomatics, Wuhan University
SBAS	Satellite-Based Augmentation System
SSR	State-Space Representation
STD	Standard Deviations
STEC	Slant Total Electron Content
TEC	Total Electron Content
TECU	Total Electron Content Unit
UT	Universal Time
VTEC	Vertical Total Electron Content
WAAS	Wide Area Augmentation System
WUHAN	Wuhan University
